# A Fatal Case of Influenza B Myocarditis with Cardiac Tamponade

**DOI:** 10.1155/2018/8026314

**Published:** 2018-08-30

**Authors:** Dominick Roto, Michelle L. Malnoske, Shira Winters, Steve N. Georas

**Affiliations:** ^1^Department of Medicine, University of Rochester Medical Center, 601 Elmwood Avenue, Rochester, NY 14642, USA; ^2^Division of Pulmonary and Critical Care, Department of Medicine, University of Rochester Medical Center, 601 Elmwood Avenue, Rochester, NY 14642, USA; ^3^Department of Pathology, University of Rochester Medical Center, 601 Elmwood Avenue, Rochester, NY 14642, USA

## Abstract

**Background:**

Influenza B is generally regarded as a less severe counterpart to influenza A, typically causing mild upper respiratory symptoms. Myocardial involvement with influenza B is a rare complication, better described in children than adults. However, when it occurs, it can lead to profound myocarditis with progression to shock requiring aggressive supportive care.

**Case Presentation:**

We present a case of cardiac tamponade in the setting of influenza B infection in a previously healthy 57-year-old woman, with progression to refractory shock and death. Autopsy revealed myocardial necrosis with infiltration of CD3+ lymphocytes, and little evidence of viral pneumonia.

**Conclusions:**

Myocarditis is a rare complication of influenza B in adults, and subsequent pericardial effusion with tamponade physiology is a previously unreported event in an otherwise healthy adult without other medical comorbidities. While rare, this is a serious and potentially fatal complication that clinicians should be aware of when evaluating a patient with suspected viral illness who is exhibiting shock physiology.

## 1. Introduction

Influenza infections are a substantial cause of morbidity and mortality worldwide. Influenza viruses infect respiratory tract epithelial cells, leading to a range of clinical outcomes ranging from mild self-limited infections to severe respiratory failure and death. The factors explaining this wide range in clinical outcomes after influenza infection are under investigation and include both host- and virus-specific factors. Severe pneumonia and respiratory failure after influenza infections are often due to overexuberant immune responses leading to neutrophilic lung inflammation and lung injury (i.e., immunopathology), but why this occurs in some patients and not others is currently not known. It is also not clear why influenza can spread to distant organs and cause tissue damage outside of the lungs.

Although myocardial involvement as a result of influenza infection has been described, this is an uncommon complication. Influenza myocarditis typically presents as a mild self-limited disease, but fulminant shock has been rarely reported [1–3]. Cardiac involvement is also more common and is better described with influenza A infections, and there are only a few case reports of cardiac involvement from influenza B infections in adults [1–4]. We present a case of influenza B infection in a previously healthy 57-year-old woman presenting with cardiac tamponade with rapid progression to refractory shock and death despite aggressive resuscitative measures.

## 2. Case Presentation

**Figure 1:**
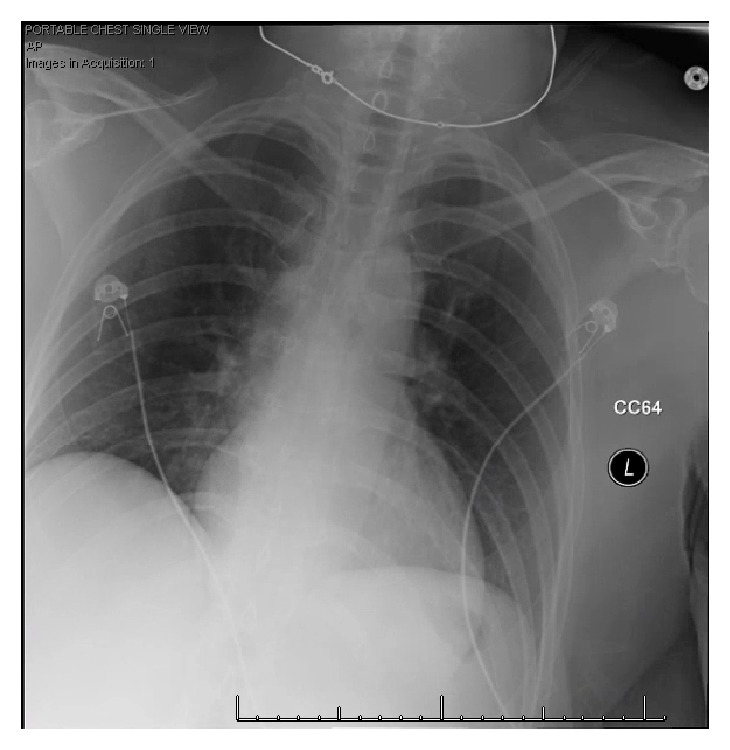
Presenting CXR showing no focal infiltrates or other evidence of cardiopulmonary disease.

A 57-year-old female without past medical history presented to the Emergency Department (ED) at the end of May with altered mental status, nausea, and vomiting. She had felt unwell for the past week with symptoms of mild cough and intermittent fevers peaking at 39.4° Celsius (C). She had been seen by her primary care physician two days prior and was diagnosed with a urinary tract infection based on a positive urine culture for Enterococcus species. She had not started the antibiotics prior to presentation to the ED. In the ED, she appeared acutely ill. She was hypotensive (blood pressure 58/41 mmHg by cuff), tachycardic (heart rate 120 beats/minute), and hypothermic (32.4°C). Physical exam revealed dry mucus membranes, clear lung fields, and cold and mottled extremities. Initial blood work demonstrated an arterial blood gas with pH of 7.0, pCO2 32mmHg, pO2 450 mmHg on supplemental oxygen, and arterial lactate 9.6 mmol/L. Chemistries and hepatic function testing showed creatinine of 1.64 mg/dL, glucose 330 mg/dL, alanine transferase 23 U/L, and total bilirubin <0.2mg/dL. Complete blood count was notable for leukocytosis 16,300/uL with 77.4% neutrophils and 16.9% lymphocytes and hemoglobin of 18.6 g/dL. CRP was normal at 2mg/L. Procalcitonin was 0.89 ng/mL. Troponin T was elevated to 0.20 ng/mL which subsequently rose to 0.97 ng/mL on repeat. Urine toxicology screen was negative. Initial chest X-ray (CXR) showed no acute cardiopulmonary disease (Figure 1). Initial ECG demonstrated sinus tachycardia. Three liters of isotonic intravenous fluids were given as bolus infusion, which resulted in transient increases in blood pressure, but systolic blood pressure remained low (<70 mm Hg) despite fluid resuscitation. A left subclavian triple lumen catheter was inserted, norepinephrine was initiated to maintain mean arterial pressure >60 mmHg, and the patient received cefepime and vancomycin for presumed septic shock.

**Figure 2:**
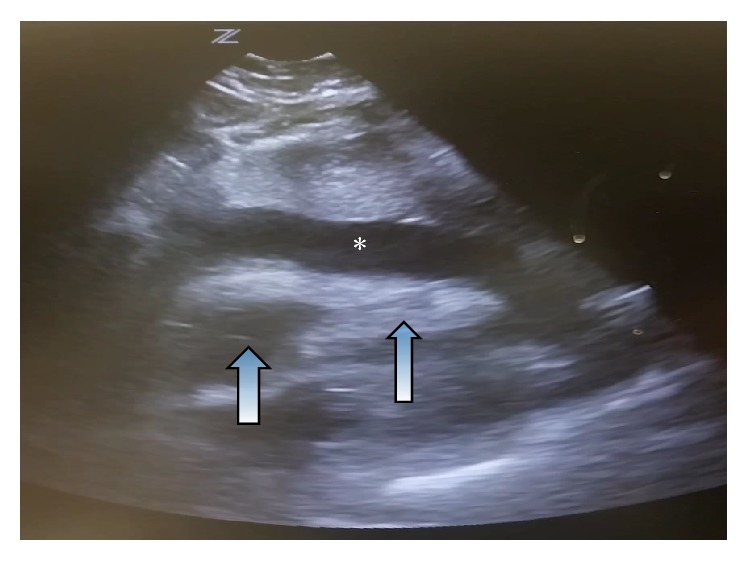
Subxiphoid window on bedside echocardiography demonstrating a large pericardial effusion (indicated by the *∗*) associated with right ventricular (RV) and right atrial (RA) collapse (indicated by the arrows).

A bedside cardiac ultrasound was performed which demonstrated a large pericardial effusion with tamponade physiology (Figure 2). The patient was taken to the cardiac catheterization lab for an urgent pericardiocentesis. Prior to the procedure, the patient suffered an asystolic cardiac arrest secondary to pump failure requiring 10 minutes of cardiopulmonary resuscitation. She was intubated and started on mechanical ventilation. The patient underwent pericardiocentes with immediate evacuation of 90ml of serous fluid, and a pericardial drain was subsequently placed to manage any ongoing or residual effusion. After successful pericardiocentesis, the patient also underwent coronary angiography, which revealed angiographically normal coronaries, with no evidence of plaque or obstruction.

The patient was admitted to the Medical Intensive Care Unit. Over the following 24 hours her condition deteriorated with hypotension and a marked metabolic acidosis despite IV fluids, high dose vasopressors, broad-spectrum antibiotics, stress dose steroids, and a bicarbonate drip. Arterial lactate continued to trend up to 16.2mmol/L. Repeat transthoracic echocardiogram 12 hours after the pericardiocentesis revealed only a small anterior pericardial effusion, with normal left and right ventricular ejection fractions. Total pericardial drain output was 150mL over this time. During this time, her respiratory viral panel taken by nasal swab on admission returned positive for influenza B by PCR. Repeat CXR showed bilateral infiltrates concerning for acute respiratory distress syndrome. Oxygenation deteriorated despite high Fi02 and PEEP, and the patient was paralyzed and started on low tidal volume ventilation. Despite maximal supportive care, later that day the patient experienced another asystolic cardiac arrest from persistent hypoxia, respiratory failure, and worsening acidosis for which resuscitative efforts were unsuccessful. All blood cultures remained negative. Urine culture from presentation returned positive for 50,000 colonies of Enterococcus species; however, the urinalysis was not compatible with active infection, showing only 12 white blood cells. Repeat urine culture collected later during the admission remained negative.

**Figure 3:**
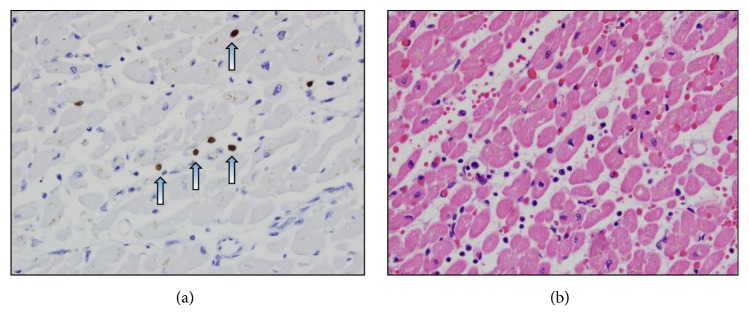
(a) Immunohistochemical stain of CD3 lymphocytes in the intraventricular septum (indicated by the arrows), 400x. (b) Hemoxyline and Eosin stain of the intraventricular septum showing hemorrhage and myocyte necrosis, 400x.

Autopsy was performed after consent was obtained from family members. On gross evaluation, the myocardium was grossly firm, dense, and mottled in appearance. Lung histology revealed relatively preserved lung architecture with only focal evidence of lung injury in the left lower lobe, but no widespread evidence of pneumonitis or pneumonia. All cultures from the lung were negative for infection. In contrast the heart was markedly abnormal, with multifocal cardiac cell necrosis and subendocardial septal hemorrhage consistent with myocarditis. The pericardium was normal. Immunohistochemistry revealed extensive infiltration of the myocardium with CD3 positive lymphocytes, and hemoxylin and eosin stain demonstrated hemorrhage and myocyte necrosis (Figure 3). The cause of death was felt to be myocarditis secondary to influenza B infection, given the strongly positive viral PCR.

## 3. Discussion and Conclusions

Cardiac complications of influenza infections have been described in the literature as early as the 1900s [4]. These complications are generally more common among those infected with influenza A. While case reports of myocarditis and subsequent shock associated with influenza B have been reported, this presentation is more common in children [3–5]. The data on these complications in adults are limited to a small number of case studies [6–9]. To our knowledge, there have not been any reports of influenza B leading to pericardial effusion and tamponade in adults.

Influenza myocarditis is traditionally defined by a viral prodrome followed by abrupt decline in cardiac function [10]. Spontaneous recovery is the most common outcome, though there have been case reports of abrupt and sudden death, as well as progression to a chronic, dilated cardiomyopathy [10–12]. Diagnosis is generally made by transthoracic echocardiogram in the appropriate clinical context, with findings typically consisting of a globally reduced ejection fraction, though often times the diagnosis is only made postmortem [13]. Histologically, myocarditis is most often associated with patchy infiltrate of the lateral free wall of the left ventricle and consists primarily of macrophages. The right ventricle is typically spared [13].

This case exhibits numerous uncommon features when comparing it to current literature for influenza-associated myocarditis. While the initial clinical presentation was consistent with a viral prodrome leading to septic shock, the development of cardiogenic shock secondary to cardiac tamponade has not been previously described. In general, infection makes up a small percentage of large volume pericardial effusions, with influenza B not being regarded as a common causative agent [14]. Moreover, infection without concurrent pericarditis is very poorly reported in the literature. In this case, there was no evidence of pericarditis on postmortem evaluation. In terms of clinical course, the patient deviated from previous described cases quite drastically in terms of physiologic presentation. While initially presenting in cardiogenic shock, the subsequent transthoracic echocardiograms showed normal cardiac function as determined by ejection fraction. Although all cultures obtained from this patient both pre- and postmortem were negative for infectious etiology, at the time of death she exhibited septic shock type physiology. Previously reported cases of influenza myocarditis generally maintain cardiogenic shock physiology, at times requiring mechanical cardiac support such as an intra-aortic balloon pump or extracorporeal membrane oxygenation (ECMO) [15]. Additionally, previously reported cases typically are self-limited, unlike the fatal course in our patient.

Treatment for influenza-induced myocarditis remains supportive. It is not known if the patient received an influenza vaccine for the season. She was not treated with antiviral therapy given her late presentation. Why exactly this patient developed such a fulminant and fatal course is not known. We suggest that clinicians remain alert for unusual complications of influenza infection for patients presenting with shock physiology, including pericardial tamponade and myocarditis.

## Abbreviations

ED: Emergency Department
C: Celsius
mmHg: Millimeters of mercury
EF: Ejection fraction
CXR: Chest X-ray.
